# Development of a digitalised complex intervention to promote activity of older people in rural areas: study protocol of a feasibility study

**DOI:** 10.1186/s40814-026-01872-0

**Published:** 2026-06-17

**Authors:** Katrin Beutner, Juliane Lamprecht, Sigrid Roggendorf, Paula Aßmann, Gundula Hübner, Anke Steckelberg, Heike Schmidt

**Affiliations:** 1 Institute of Health and Nursing Science, Martin Luther University Halle-Wittenberg, Halle (Saale), Germany; 2 Institute of Psychology, Martin Luther University Halle-Wittenberg, Halle (Saale), Germany; 3 Department of Psychology, MSH Medical School Hamburg, University of Applied Science and Medical University, Hamburg, Germany; 4 Institute of Health and Nursing Science, Medical Faculty, Martin Luther University Halle-Wittenberg, Magdeburger Straße 8, Halle (Saale), 06112 Germany

**Keywords:** Complex intervention, Physical activity, Elderly, Rural areas, Digital assisted, Participatory research, Physical therapy, Participation

## Abstract

**Background:**

Age-related mobility limitations are associated with reduced activity and loss of participation, which can result in loneliness and depression. Physical activity programmes can help maintain the functional status and quality of life of older individuals, but they are rarely available and often difficult to reach, especially in rural areas. Digital-assisted programmes have the potential, independent of place and time, to activate and promote physical function and social participation in older people. Therefore, this study aims (1) to develop a complex intervention comprising digital elements to promote physical activity, quality of life and participation in older people with impending or existing functional limitations and (2) to test the feasibility, acceptability, and potential benefits of the developed intervention.

**Methods:**

The development and piloting of the intervention follow the UK Medical Research Council framework for complex interventions. The intervention will be developed through participatory co-production, integrating the perspectives of all potentially relevant user groups, including older people, physiotherapists, general practitioners, and day care facilities in the participating regions. Co-production is guided by the principles of early stakeholder involvement, iterative refinement, usability-oriented development, and shared decision-making regarding intervention content and delivery. The developed intervention will be examined using a consecutive control intervention group design with 60 older people (aged ≥ 65 years) in rural areas of Saxony-Anhalt who have existing or imminent mobility restrictions and have a medical prescription for physiotherapy. The primary feasibility outcomes will be recruitment rate, completion rate, acceptability, and compliance with the study procedures and the intervention assessed by protocols, utilisation data, questionnaires, and interviews at 20-weeks follow-up. Quantitative and qualitative findings will be integrated during interpretation using a convergent mixed-methods approach. Secondary patient-related outcomes will be physical function, physical activity, self-care, quality of life, and participation measured at baseline and after 12 weeks.

**Discussion:**

The study aims to evaluate the feasibility of a new intervention designed to promote physical activity and participation among older people living in rural areas. The findings will inform intervention refinement and the design of a future definitive randomised controlled trial.

**Trial registration:**

DRKS, DRKS00031574. Registered on 5 May 2023, https://drks.de/search/de/trial/DRKS00031574.

**Supplementary Information:**

The online version contains supplementary material available at 10.1186/s40814-026-01872-0.

## Background

Increasing life expectancy poses new challenges for the provision of healthcare to the elderly, particularly in rural areas. The needs of an aging society are growing while capacity is limited e.g. due to a shortage of skilled healthcare professionals. Ageing is typically linked to a loss of function [1], but biological age, functional capacity and the number and severity of chronic diseases vary widely. Physical activity (PA) has positive effects on maintaining and improving functional status and quality of life [2, 3]. Maintaining PA and physical performance is crucial for preserving independence and self-care, especially in older age [4–6]. Independence and autonomy, as well as social contacts, have a positive impact on quality of life. Reduced PA and loss of social participation due to mobility limitations can lead to loneliness and depression [6, 7]. PA programmes can assist in maintaining the functional status and quality of life of older individuals [8, 9]. However, these programmes are rarely available and often difficult to access, especially in rural areas.

Programmes for independent activation often require individuals to possess health literacy, digital literacy, and personal initiative to select and carry out the exercises. Regarding the variety of physical function, comorbidities and respective limitations in older adults, it is advisable to combine such programmes with professional advice. Assisted living [10] and digital interventions to promote physical function [11–16] as well as cognitive ability and social participation in older people are being discussed as potential solutions [17, 18]. Interventions that include instructions, exercise plans, and elements for enhancing motivation, such as activity monitoring or reminder functions [12], should be distinguished from game-based interventions only [15, 16]. Individual planning, goal setting, and targeted recommendations appear to increase the effectiveness of digital programmes [12, 19, 20]. Furthermore, research suggests that exergaming programmes have a positive impact on mobility and balance [21, 22] as well as cognitive function when compared to physical training [13, 23]. Motivation, adherence and enjoyment also appear to be higher with exergaming activities [21, 24, 25]. Additionally, frail older individuals may benefit from digital healthcare interventions [26], provided that the technical requirements are met [27]. Furthermore, it is crucial to consider the usability barriers that have been identified in the design and development phase of a digital intervention for elderly users [28]. These barriers include font size and style, screen size, colour contrast, complex interface and functions, visual feedback of operations, lack of knowledge and experience, and menus with too many options and navigation issues. It is important to address these barriers to ensure a user-friendly experience for elderly users.

However, evidence on digital programmes for maintaining PA and social activation is lacking due to methodological heterogeneity. Current studies have shown that older women with impending mobility restrictions (pre-frail) have a higher participation rate in the home training compared to group training in the study centre with comparable effectiveness in terms of physical function [29]. Additionally, multi-component videogame-based training has been found to have high training participation, good usability, and a positive impact on physical and cognitive function [30].

Apps for elderly fitness and exercise offer a wide range of exercise options, including seated gymnastics, cardio training, coordination exercises, and yoga. This suggests a broad interest among the target group and high demand in the market. While tracking allows for external and self-monitoring of exercise quantity, no app can control the quality of exercise or if the exercises are suitable for the physical limitations. Only a few apps provide the option to contact a coach or therapist for assistance. This is typically a virtual coach rather than a real therapist. Gaming applications and support for social contacts have not yet been integrated.

To promote physical function and social participation in older people, specific primary, secondary and tertiary prevention programmes are necessary. It is important to consider the heterogeneity of this target group to maintain their autonomy and self-care.

### Objectives

The study aims to (1) develop an interprofessional complex intervention comprising digital elements to promote PA, quality of life and participation in older people with impending or existing functional limitations and (2) test the feasibility of the developed intervention.

The intervention aims to motivate and empower participants to exercise independently at their own home through individually tailored exercise programmes. In addition, the intervention will initiate and support digital social participation, including internet use and digital communication. The feasibility study aims to evaluate feasibility, acceptability, and potential benefits of the intervention components from the perspectives of patients, general practitioners, and physiotherapists and to explore barriers and facilitators for implementation. Specifically, the evaluation will focus on the following components:

- Individual exercise programmes,
- Complementary gaming applications,
- Access to the digital intervention,
- Digital options for social activation.
- Monitoring by physiotherapist via video therapy.

## Methods/design

### Design

Following the UK Medical Research Council (MRC) framework for the development and evaluation of complex interventions [31, 32], the study design included a stepwise approach with three phases:

- 1st phase: development of the intervention using participatory, co-creative processes with all potentially relevant user groups, e.g. older people, physiotherapists, general practitioners, and day care facilities in the participating regions,
- 2nd phase: pre-testing of the developed intervention
- 3rd phase: feasibility testing of the intervention with a consecutive usual-care control intervention group design in the healthcare context, including evaluation of acceptability, implementation, and potential benefits from the perspective of all participants using a convergent mixed-methods approach.

This study is primarily designed as a feasibility study rather than a pilot randomised-controlled trial. The main purpose is to assess recruitment, retention, intervention delivery, acceptability, adherence, usability, data-collection procedures, and implementation barriers and facilitators. The study is not designed to assess the feasibility of randomisation.

**Fig. 1:**
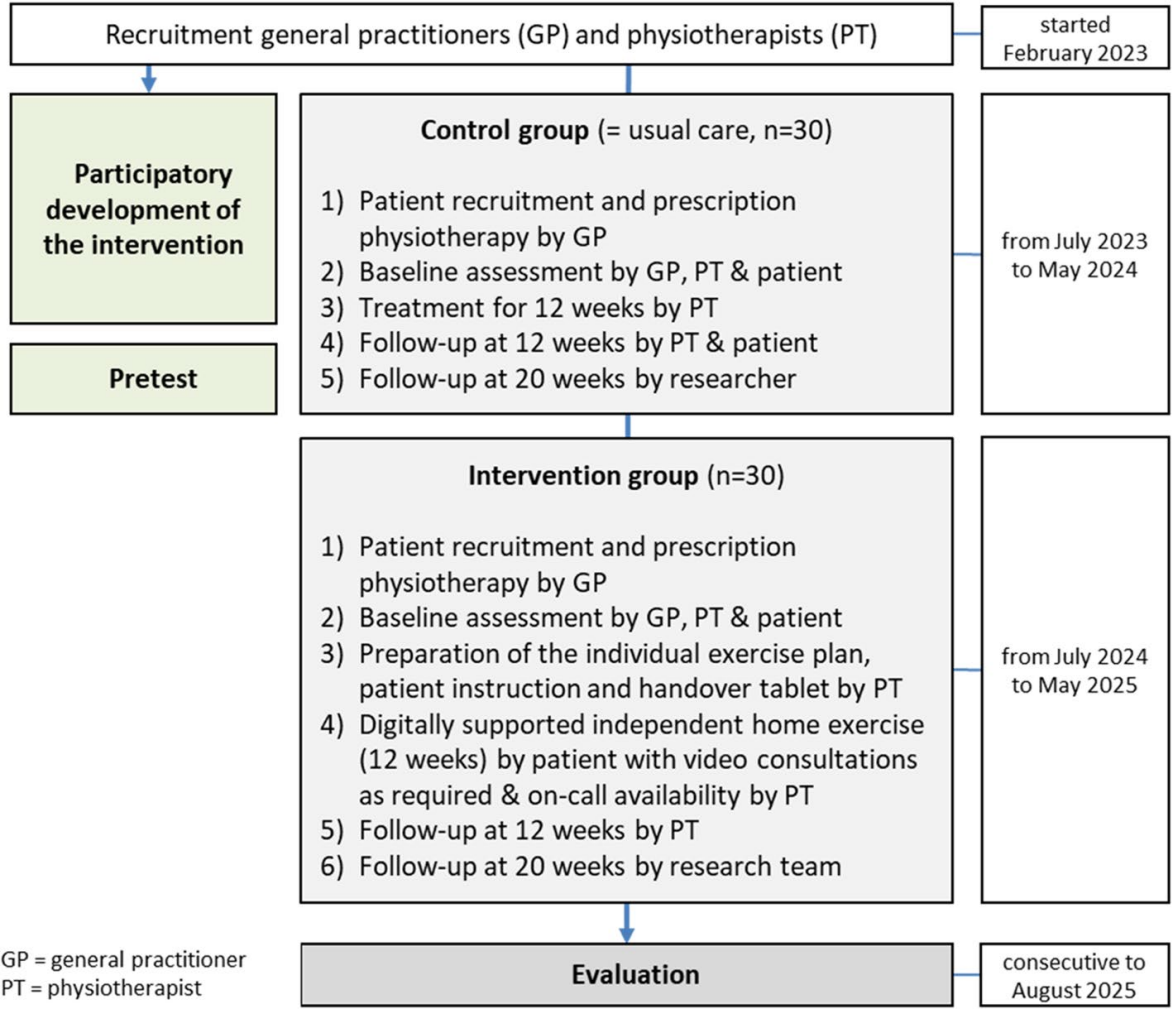
Flowchart of the study course

During the 1 st phase (intervention development), the recruitment, delivery, and evaluation of the control group (usual care) are performed simultaneously (see Fig. 1). The control group was included to provide contextual information on usual care, study procedures, and change trajectories over time in a comparable population. It is not intended for confirmatory hypothesis testing or formal between-group effectiveness comparisons.

Even though the control group is conducted and assessed alongside the intervention development, the results for the intervention group are not expected to be significantly confounded due to the distinct nature of the intervention. While the control group includes standard physiotherapy on a face-to-face basis per appointment, the intervention will focus on independent digitalised home-based exercise supported by video consultations. In addition, the measurements will remain the same but will include additional measures related to tablet usage.

### Intervention development and pretest

This study is based on a previous project that developed an individually tailored unsupervised physical activity programme to promote physical function in older cancer patients [33]. As maintaining physical function is a crucial objective for all older individuals, the intervention will be expanded to target all elderly people who face difficulty accessing community activity programmes due to limited functional capacity and a lack of infrastructure in rural areas. A low-threshold, easily accessible, tablet-based digital intervention will be developed to provide individually tailored incentives for sustainable activation and maintenance of social contacts, based on professional advice. Particular attention will be paid to ease of use and acceptability, even for patients with no previous experience of using a digital device.

The intervention will be developed and piloted using the results of the previous project. Specifically, the exercise portfolio will be expanded, the videos will be optimised, and the assessment will be adapted to the new target group. The exercises will be supplemented with complementary gaming applications to increase the motivation to exercise independently. The gaming applications are controlled by the participants’ movements, combining physical activity with entertainment. The intervention application will be stored on a tablet and transferred to the television via a cable by the participants. The tablets will be equipped with an adapted interface for older people. If necessary, an alternative control element (SmartCard) [34] will be provided to ensure that participants without any technical experience can also use the devices. Furthermore, each tablet will contain applications that encourage social interaction.

The development of the intervention will be based on a co-production approach. The co-production refers to the active involvement of future users and relevant implementation partners throughout intervention design, with the aim of ensuring relevance, usability, acceptability, and contextual fit. The co-production process is guided by four principles: (1) early and continuous stakeholder involvement, (2) valuing experiential and professional knowledge equally, (3) iterative refinement of intervention components, and (4) joint reflection on implementation requirements in routine care.

To operationalise these principles, we will conduct a series of participatory workshops with older people from the participating regions. We anticipate approximately 3–5 workshops with 6–10 participants per workshop, depending on the need for further refinement and whether thematic saturation is achieved. Participants will be purposively approached to ensure diversity regarding age, gender, living situation, prior digital experience, and degree of functional limitation. Recruitment will take place via participating general practices and physiotherapy practices, day care facilities, and regional cooperation partners.

The first workshop(s) will explore needs, barriers, and facilitators related to physical activity, social participation, use of digital technology, and support needs in everyday life. Subsequent workshop(s) will focus on discussing and prioritising desired intervention functions, reviewing first prototypes or mock-ups, and testing initial intervention components such as exercise videos, interface design, gaming applications, and options for digital social interaction. Later workshop(s) will be used to refine content, language, usability, and delivery procedures. Workshop methods will include moderated group discussion, feedback on visual and functional prototypes, simple ranking and prioritisation tasks, and structured field notes by the research team.

The views of general practitioners and physiotherapists will be explored through focused consultations and qualitative interviews addressing perceived patient needs, feasibility of recruitment and referral pathways, intervention relevance, required support materials, and implementation in routine care. Their perspectives will be incorporated iteratively into intervention development, particularly regarding exercise selection, monitoring procedures, and integration with usual care. Day care facilities will contribute perspectives on usability, implementation context, and opportunities for social activation.

Involving potential users, especially older patients and physiotherapists, from the outset of the project can ensure a high level of acceptance and applicability of the intervention.

The pretest will focus on usability and involve older individuals, physiotherapists, and day care facilities in the participating regions. The pretest is conceived as an iterative usability phase. We plan to begin with approximately 3–5 older adults and continue testing iteratively, if necessary, until no substantial new usability issues emerge. Where feasible, a heterogeneous patient sample will be selected to reflect a range of technical experience, motivation, and physical limitations.

The usability test will be conducted both in the home environment and, where appropriate, with one cooperating day care facility recruited through existing cooperation partners. For the pretest, observations will be combined with the think-aloud method. During the pretest, participants will be observed and asked to verbalise their thoughts while interacting with the intervention. This approach enables the physiotherapist and researcher to gain insights into the participants’ current experiences, thoughts, questions, and emotions, which can help identify usability issues. Additionally, participants will rate the technical components using a traffic light system and record expected benefits. Think-aloud sessions will be audio-recorded, documented in structured observation protocols, and analysed using a focused qualitative content analysis with an explicit usability orientation. Identified problems will be categorised, for example, as relating to navigation, comprehension, physical handling, visual design, motivation, or technical barriers. Findings from each pretest round will directly inform iterative refinement of the intervention before the start of the main feasibility phase.

### Testing of the complex intervention

A feasibility study with embedded pilot component will be conducted to test feasibility, acceptability, and potential benefits of the developed intervention with 60 elderly patients in a consecutive control intervention group study in the healthcare context. The evaluation will be conducted using the framework provided by Moore et al. [35] to assess feasibility by measurement of recruitment rate, completion rate, acceptability and compliance with the intervention and to examine the factors that facilitate or hinder the implementation of the intervention.

#### Participants and recruitment

##### Setting and eligibility criteria

The study will be performed with general practitioners and physiotherapists in rural areas of Saxony-Anhalt. We will include patients aged 65 years or older with impending or existing functional limitations, who live in their own homes in the district of Harz or Burgenlandkreis, who can move around independently (with or without mobility aids), and who can understand and speak German. The impending or existing functional limitations will be diagnosed by general practitioners by assessing aspects of cognition, motor skills, mood, and participation/social situation. Patients with severe dementia, acute mental illness, bed confinement, severe impaired vision/reading ability, legal guardian, and contraindications for activity will be excluded.

##### Recruitment of general practitioners, physiotherapists, and study participants

General practitioners and physiotherapists will be recruited from convenience samples of interested or existing cooperation partners, from online business directories of the participating districts, and through cooperation with professional associations. They will receive an invitation to participate in the study via letter or email, followed by a telephone call, as well as through information events (both online and face-to-face). The researchers will present the study to interested practitioners and provide them with detailed information. Upon providing a written informed consent, they will be enrolled in the study.

The general practitioners will identify eligible patients and provide them with oral and written study information in order to invite them to participate in the study. Researchers will be available to answer questions or provide additional information in person or by phone. Eligible patients will be asked to provide informed consent, which they may withdraw at any time without giving reasons and without any disadvantages for the patients. The general practitioners receive an expense allowance per included patient.

#### Sample size calculation

A formal sample size power calculation is not appropriate for this feasibility study as the outcomes were the feasibility and acceptability of the intervention rather than effectiveness [36]. Accordingly, the sample size was chosen primarily to estimate key feasibility parameters with acceptable precision and to inform the design of a future definitive trial. The selected sample size of 60 patients, including 30 participants in the intervention group, is consistent with published recommendations for pilot and feasibility studies [37–39]. For example, a sample of 30 participants in the intervention group allows estimation of key proportions such as adherence, intervention uptake, and study completion with sufficient precision to judge whether predefined feasibility criteria are likely to be met and to identify major implementation problems. The chosen sample is therefore intended to support estimation of recruitment, retention, adherence, acceptability, and data completeness, rather than formal hypothesis testing. A review of studies on data saturation in qualitative research indicates a sample of 9–17 interviews as sufficient to achieve saturation [40]. Therefore, the selected sample of 60 patients should provide sufficient information about the feasibility and acceptability of the intervention.

As physiotherapists are already characterised by a high workload, we assume that no more than two additional study patients per therapist can be treated at the same time. In this respect, a sample size of 30 per group also corresponds to the maximum number of patients that could be treated by five physiotherapists within 39 weeks of the intervention period of each group. It is anticipated that each general practitioner will be able to recruit two patients per month per study group over the course of a 5-month recruitment period. Consequently, two general practitioners will be required per district, with the objective of recruiting 30 patients per study arm. In order to achieve an adequate number of participants, we will regularly monitor the response rate and, if necessary, consider expanding the districts in which recruitment takes place.

#### Blinding

Due to the characteristics of the intervention and the study design, it is not possible to blind the general practitioners, the physiotherapists, the patients or the researchers involved in delivering the intervention to the group allocation.

#### Control group

The control group is included to describe usual care, to assess feasibility of recruitment and data collection procedures in a comparable population, and to provide contextual information for interpreting change over time. The study is not powered for confirmatory between-group comparisons, and the control group should therefore not be interpreted as part of a definitive trial.

The recruitment, delivery, and evaluation of the control group (usual care) occur simultaneously during intervention development (= 1 st study phase; see Fig. 1). Participants in the control group will receive a study-specific prescription for physiotherapy from their general practitioner. The baseline assessment (T0) will be recorded during the first physiotherapy session. After this, physiotherapy treatment (as usual) will take place according to the prescription, with one session per week for a maximum of 12 weeks. The physiotherapists receive an expense allowance per patient.

Participants who do not yet use their own digital devices are offered options for social activation through loan tablets. The tablets provide contact options with relatives, friends or neighbours via phone, video call, email, or text-messaging programmes, and come equipped with an adapted interface for older people. If necessary, an alternative control element (SmartCard) [34] will be provided to ensure that participants without technical experience can also use the devices.

#### Intervention group

The recruitment, implementation, and evaluation of the intervention group will be started after the development and pre-testing of the intervention has been completed (= 3rd study phase; see Fig. 1). Participants in the intervention group also will receive a study-specific prescription for physiotherapy from their general practitioner. The baseline assessment (T0) will be recorded during the first physiotherapy session. After this, physiotherapy treatment will take place as a hybrid model for a maximum of 12 weeks. A hybrid model refers to a combination of online physiotherapy sessions (video therapy) and independent home exercises by the patients, with an individual digital exercise plan, based on a physiotherapeutic assessment. The physiotherapist and patient collaboratively will select exercises from a diverse portfolio to create an individualised exercise programme. The portfolio consists of exercises designed to improve coordination, balance, mobility, strength, and endurance. The exercises can be performed in a sitting or standing position, or on the floor, and are available at different difficulty levels. The physiotherapist will transfer video-based instructions for the exercises to the patient’s tablet. The selected exercises will be practiced face-to-face under therapeutic supervision during an appointment, either in the practice or at home. Patients will then carry out the selected exercises independently at home for the following 12 weeks. The physiotherapist and the patient will jointly determine the frequency of further physiotherapy sessions using video therapy (20 min each). The online sessions will be utilised to address concerns or questions, demonstrate proper exercise techniques, or discuss adjustments of the exercise plan. Video-based instructions and movement monitoring, which recognises body gestures and postures, will aid in the accurate execution of exercises. Also, face-to-face sessions will be available if needed. The physiotherapists receive an expense allowance per patient.

Game applications will be designed to promote motivation to exercise and sustainable activation. The games can be controlled by recognising poses through the tablet’s camera and include everyday movements. To ensure compliance with data-protection regulations, physiotherapists will receive funding for certified video therapy software during the study period. All patients of the intervention group will be provided with a tablet that grants access to the intervention and social activation options, similar to the control group.

## Outcome measures, data collection, and data management

###  1 st phase – intervention development

Information recorded will include gender, age, place of residence (urban vs. rural), and usage of digital devices and the internet for all workshop participants, as well as the respective healthcare professionals’ professions.

### 2nd phase - pre-testing of the intervention

The pretest of the intervention (usability test) will be based on observations of the participants using the think-aloud method. We will document observations in an observation protocol, which will record the comments made as part of the think-aloud process. The gender, age, and previous digital experience of the participating patient and physiotherapist will also be recorded. A digital audio recording will be made with the consent of all participants. Furthermore, participants will rate the technical components using a traffic light system and record expected benefits.

### 3rd phase - pilot study and evaluation

During piloting of the intervention, data for outcomes and other variables will be collected continuously (e.g. recruitment rates, content and duration of physiotherapy and tablet use) or at three measurement points: at baseline (T0), after 12 weeks (T1, end of physiotherapy treatment), and after 20 weeks (T2, follow-up). An overview of the different primary and secondary outcome variables is provided in Tables 1 and 2.

**Table 1:** Primary feasibility outcomes

Outcome	Definition/measurement	Data source	Success threshold
Recruitment rate patients	Proportion of patients approached who meet the eligibility criteria and agree to enter the study; Reasons for agreement or disagreement	Recruitment protocol general practitioner; interview with patients and general practitioner at T2^c^	> 50%
Recruitment rate professionals	Proportion of physiotherapist and general practitioners approached who agree to enter the study; Reasons for agreement or disagreement	Recruitment protocol study team; interview with physiotherapists and general practitioner at T2^c^	> 30%
Completion rate	Proportion of all study patients that remain participants until the end of the defined study period	Study management protocol	≥ 95%
Compliance with the intervention	Proportion of all patients who receive a scheduled, individually digital exercise plan	Documentation physiotherapist	100%
	Proportion of all patients who used the tablet for exercise at least one day per week; content and duration of each tablet usage	Tablet user data	≥ 80%
	Proportion of all patients who used the tablet for gaming at least one day per week; content and duration of each tablet usage	Tablet user data	≥ 80%
	Proportion of perceived video calls that were suggested by the PT; content and duration of each physiotherapy session	Documentation physiotherapist	≥ 80%
Compliance with study procedures	Proportion of complete data	Study management protocol	≥ 90%
	Proportion of participants who rate assessments as feasible to use	Interview with patients, physiotherapists and general practitioners at T2^c^	≥ 80%
	Proportion of participating professionals who rate the study procedures as feasible		≥ 80%
	Proportion of participants who use the supporting materials provided by the study team		≥ 80%
Acceptability	Views and experience of the intervention, amount of perceived barriers and facilitators	Interview with patients, physiotherapists and general practitioners at T2^c^	Not applicable
	Attitude and experience with modern technology	Patient questionnaire at T0^a^, T1^b^ and interview at T2^c^	

^a^*T0*: baseline assessment

^b^*T1*: 12-week follow-up (end of physiotherapy)

^c^*T2*: 20-week follow-up

**Table 2:** Secondary patient-related outcomes

Outcome	Instrument	Data source	Measurement point
Balance, walking speed, strength of the lower extremities	Short Physical Performance Battery, SPPB [42]	Physiotherapist	T0^a^, T1^b^
Physical activity in everyday life and sporting activities, including facilitators and barriers to physical activity adherence, and the intention to carry out regular physical activity (at least 30 min on at least 5 days a week)	Freiburg Physical Activity Questionnaire, FFB [46] and additional items	Patient-reported via questionnaire (T0, T1) or interview (T2)	T0, T1, T2^c^
Activities of daily living	Hannover Functional Ability Questionnaire (FFbH) [47]		T0, T1
Quality of life and well-being	Short-Form Health Survey, SF-8 [43]		T0, T1
Social situation and social contacts	Social Situation (Part 1), SoS [45]		T0, T1
Falls and fall-related treatment	Fall assessment, self-generated items		T0, T1

^a^*T0*: baseline assessment

^b^*T1*: 12-week follow-up (end of physiotherapy)

^c^*T2*: 20-week follow-up

#### Primary feasibility outcomes

The primary aim of the study is to assess the feasibility and acceptability of the intervention and the study procedures. Therefore, the primary feasibility outcomes are recruitment rate, completion rate, acceptability, adherence, utilisation, and compliance with study procedures, including time requirements, resource demands, data collection, data management, and the usefulness of supporting materials (see Table 1).

In addition to the continuous recording of recruitment rates, including reasons for participation or non-participation, structural data of participating practitioners and physiotherapists will be collected, including the location of the practice (urban vs. rural), the number of employees (< 5, > 5), the average number of patients per week, the proportion of older patients (65 years or older), and experience with digitalisation (scheduling, documentation, use of video therapy, or electronic patient records). Furthermore, patients’ technical requirements are documented, including the presence of a television and a PC or tablet, access to the Internet, and technical experience.

Acceptability and compliance with the intervention and the study procedures will be assessed with patients, physiotherapists, and general practitioners taking part in the study by obtaining their views and experience of the intervention in interviews. Qualitative interviews will be conducted with all participants from the intervention group, as well as all participating physiotherapists and general practitioners. The interview guides will address experiences of intervention use, perceived relevance and burden, usability of the technical components, experiences with video therapy, and perceived changes in physical activity and social participation. We will assess the level of intervention implementation and associated effort, as well as the acceptability of the intervention to both patients and healthcare practitioners. Furthermore, an evaluation of the helpfulness of the provided support materials will be conducted, including an assessment of the exercise overview for therapists, exercise videos, written instructions for selected exercises, and the user manual for patients. Additionally, we will examine factors that facilitate or hinder the implementation of the intervention, short- and long-term adherence, and behavioural change. The qualitative evaluation will be informed by behaviour change and implementation perspectives. Interview guides for patients and healthcare professionals will draw on UTAUT [42] and on implementation-related questions concerning capability, opportunity, and motivation to engage with the intervention and associated procedures. This theoretical orientation will support both data collection and analysis of behavioural and implementation-related barriers and facilitators.

Compliance with the intervention also will be assessed by analysing the physiotherapy sessions and tablet usage. For each tablet use, user data on the frequency and duration of use, as well as the services utilised, will be conducted. This comprises the use of the intervention elements, including exercises and gaming applications, as well as options for social contact. In addition, all face-to-face and online physiotherapy sessions will be documented on the physiotherapy prescription, detailing the duration, content, quality and any problems encountered during each session. No content analysis of data, such as browsing history, or image data from online sessions will be carried out. As part of the patient questionnaire, different items, based on the Unified Theory for Acceptance and Use of Technology (UTAUT) [41], will be used to measure patients’ attitude and experience with modern technology.

#### Secondary outcomes

Secondary patient-related outcome measures (T0, T1) are objective and subjective assessments of physical function, physical activity, cognitive function, self-care, quality of life and participation (see Table 2).

Physical function will be measured by the SPPB at baseline (T0) and at the end of the physiotherapy after 12 weeks (T1). The SPPB [42] comprises three tests by the physiotherapist: tandem stand, walk over four metres, and sit-to-stand test. Therefore, the SPPB measures deficits in mobility, muscle strength of the lower extremities and balance. The assessments of physical activity will include self-reported physical activity in everyday life and sporting activities [32], as well as facilitators and barriers to physical activity adherence, and the patient’s intention to carry out regular physical activity (at least 30 min on at least 5 days a week). Further, physical function and activities of daily living will be measured [33]. The Short-Form Health Survey (SF-8) [43, 44] will be used to measure health-related quality of life. Each of the eight dimensions of the SF-36 is represented by a single item in the SF8. The Social Situation Questionnaire of Nikolaus [45] will be used to record social situation and social contacts through several items. This includes existing social contacts and support, and social activities.

#### Additional measures

Descriptive data will be collected at baseline, including patient-reported socio-demographic information (such as age, gender, education, and living situation), data on infrastructure or previous physiotherapy in the past 6 months, and medical data reported by general practitioner (such as body weight, body height, diagnoses (ICD-10), and limitations in cognition, motor skills, mood, and participation/social situation, reason for prescribing physiotherapy).

#### Data management

All assessments at baseline (T0) and after 12 weeks physiotherapy (T1) will be performed on a paper-and-pencil basis by general practitioners, physiotherapists and patients. The assessment at 20-week follow-up (T2) will be conducted through semi-structured guided interviews by trained members of the study team, either by telephone, video call, or face to face, depending on participants’ preferences and practical considerations.

After each measurement point, the pseudonymised patient data will be transferred to the study centre via fax or upload to a secure project cloud. A copy of the questionnaires will be safely stored at the respective practice of general practitioners or physiotherapists. The pseudonymised data will be stored on a secure computer and will be handled in accordance with the data protection declaration. All hard-copy printouts will be destroyed once the analysis is complete.

The study team will perform data entry and analysis. All data will be entered into a database and validated through double entry. Additionally, the data will be checked for inconsistencies and completeness, and its quality will be reported to the general practitioners and physiotherapists. Data quality (e.g. proportion of missing values) and organisational or logistic issues will be reviewed and discussed in weekly team meetings. In this way, necessary actions to improve data quality and study logistics can be taken in good time. In addition, training on data entry will be carried out to ensure data quality.

## Data analysis

### Primary feasibility outcomes

The recruitment rate, completion rate, and compliance with the intervention (intervention days, content and duration of each physiotherapy session and tablet use) will be described using descriptive statistics. Descriptive data analysis included the determination of absolute and percentage frequencies, medians, means, minimum and maximum values, standard deviations, and 95% confidence intervals using the commercial software IBM SPSS Statistics 25.0.

To assess feasibility, the defined criteria for determining success (s. Table 1) will be used in combination with the feasibility assessment criteria from Thabane’s framework [48]. The feasibility milestones were defined pragmatically based on prior project experience, organisational capacities in the participating regions, and values considered acceptable in comparable feasibility contexts. They are intended to guide interpretation of feasibility rather than to function as rigid pass-fail criteria. The final decision regarding the overall feasibility rating of the study protocol will be based on pragmatic considerations of the feasibility of the protocol in terms of recruitment, data collection, and the impact on professionals’ workflows. In addition, the judgment of the research team will be taken into account, which will encompass an assessment of the capacity to influence individual barriers and problems, for example through counselling or training.

Acceptability and individual compliance with the intervention, assessed through interviews at the 20-week follow-up, will be analysed using content analysis, which comprises a combination of inductive and deductive procedures, according to Mayring [49]. The interviews will be audio recorded and transcribed verbatim for analysis. MAXQDA (VERBI software, Berlin, Germany) will be used for transcription and analysis. The primary focus of the qualitative data analysis will be on answering the central research questions. It will cover general experiences and user behaviour related to the new intervention, problems and areas for optimisation, changes in physical activity, and changes in interaction and social participation. The analysis will be conducted by two independent members of the research team.

Qualitative findings will also be used to explain and contextualise quantitative feasibility results, such as low adherence, incomplete data, or barriers and facilitators for implementation. Integration of qualitative and quantitative findings will take place at the interpretation stage through joint comparison of results across feasibility domains (e.g. recruitment, intervention uptake, acceptability, and adherence). This convergent mixed-methods approach will enable a more comprehensive assessment of feasibility than either data stand alone.

### Secondary outcomes

The secondary patient-related outcomes and additional measures also will be described using descriptive statistics. Exploratory analyses of potential benefit will focus primarily on within-group change over time and descriptive comparison of change patterns across groups. The main purpose of these within-group comparisons is to determine whether the selected assessments are suitable for capturing changes in outcomes over time and to assess the preliminary patterns of change of the novel intervention prior to testing it in a main trial. The data will also provide information to estimate variability.

Any inferential analyses will be interpreted cautiously and as hypothesis-generating only. Additionally, exploratory inferential statistical data analysis will be used, including mean comparisons, correlations, and regressions to identify and describe patterns. The chi-square (χ^2^) test will be used to compare categorical variables. An odds ratio with a 95% confidence interval will be calculated as an effect measure.

Given the small sample size, formal subgroup analyses are not planned. At most, descriptive exploration of selected participant characteristics may be undertaken if this appears useful for interpreting feasibility outcomes. Missing data will be reported descriptively.

Given the nature of the intervention, we do not expect serious adverse events. Thus, no interim analysis is planned, and no stopping rules will be applied as regards safety issues. There is also no necessity for a data monitoring committee.

## Quality assurance

The study will be planned, implemented and evaluated in accordance with the principles of good clinical practice [50] and the Declaration of Helsinki [51]. Following ethical approval, the study was registered with the publicly accessible German Clinical Trials Register (DRKS) under the ID DRKS00031574 before any study participants were enrolled. This study protocol will be published in a peer-reviewed journal and it will follow the most recent Standard Protocol Items: Recommendations for Interventional Trials (SPIRIT) statement [52] and the applicable criteria from the extension of the Consolidated Standards of Reporting Trials (CONSORT) for randomised pilot and feasibility trials [36]. The corresponding checklists are presented in Additional files 1 and 2.

A detailed quality assurance plan will be developed to ensure safety. This plan will summarise the monitoring methods for data collection, validation and reporting, and will include SOPs to provide research staff, general practitioners and physiotherapists with instructions for data collection, handling, reporting and analysis.

To ensure wide dissemination, we will publish the study results in peer-reviewed scientific journals and present the results at scientific conferences. All results will be reported in accordance with appropriate reporting guidelines to enhance the quality and transparency of our research.

## Ethical and legal considerations

The research team will be responsible for ensuring that research participants have the opportunity to address any sense of burden and maintain their well-being. Any changes to the study protocol, expected or unexpected serious events, or early termination of the study will be promptly reported to the ethics committees by the principal investigators.

Participating patients are covered by insurance, as there is a risk of an unintentional fall due to independent exercise at home. However, the selected exercises will be according to the physiotherapeutic assessment and the patients’ resources, which help to minimise the risk of falling. Before the patients carry out the selected exercises independently at home, they will practice them face-to-face under therapeutic supervision during an appointment. Patients also will receive information on safety measures.

All participants will be informed that study-related data will be stored in pseudonymised form and will only be used for scientific data analysis. The study data will be kept in a locked archive for 10 years at the study centre after completion of the trial and will then be deleted. Additionally, the EU General Data Protection Regulation will apply, and all participants will be explicitly informed about their rights.

## Discussion

This study protocol describes the development and piloting of a complex intervention that aims to promote physical activity, quality of life and participation in older people with impending or existing functional limitations in rural areas. With the emphasis on maintaining physical activity as crucial for preserving independence and self-care, this intervention will address health outcomes that are highly relevant to the older individuals.

As an intervention, a prescribable “hybrid model” is being tested for feasibility and acceptance in the real healthcare setting. A hybrid model refers to a combination of online physiotherapy sessions (video therapy) and independent home-based exercises by the patients following an individual digital exercise plan, based on a physiotherapeutic assessment. The hybrid model can be an alternative to on-site physiotherapy, especially in rural areas with very old people and long distances to physiotherapy.

This study is primarily intended to determine whether the intervention and study procedures are feasible and acceptable in routine care and how the intervention may need to be refined before a future definitive trial. Although a consecutive control group is included, the study is not powered to test effectiveness between groups. Rather, the control group serves to contextualise feasibility findings and outcome trajectories under usual care.

The intervention addresses three fields of action: (1) primary prevention: general physical activation for the targeted prevention of loss of function, falls and osteoporosis, as well as social activation to prevent loneliness in old age and depression; (2) secondary prevention: targeted promotion of coordination, balance, mobility, strength and endurance in the case of existing limitations or reduced function; and (3) continuation of training in the home environment following inpatient or outpatient rehabilitation in order to achieve better sustainability of the effects.

Developing complex interventions is a challenging task. Therefore, our study underwent a careful intervention development process that included the proposed core elements in the updated MRC Framework for the development and evaluation of complex interventions [32]. These elements are identifying the context, engaging relevant stakeholders in the development process, creating a logic model, and considering the cost perspective. A particular strength of the present study is the explicit co-production process with older people and healthcare professionals, combined with usability pretesting and mixed-methods feasibility evaluation.

By using this methodological approach that includes pre-testing and piloting in a real healthcare environment, as well as evaluating patient-relevant outcome measures and combining quantitative and qualitative analyses, we expect to obtain reliable results regarding the feasibility, acceptability and potential benefits of the developed intervention in terms of social and physical activation. The findings will be used to refine intervention components, optimise implementation procedures, and inform the design of a future randomised controlled trial.

Thanks to the close cooperation between science, technology, and service providers, the project has the potential to make an important contribution to the further development of healthcare for older people in the region in order to secure and recruit skilled workers. In future, the intervention could be prescribed by the physician and, due to its independence of time and place, close a gap in the healthcare system.

## Trial status

At the time of manuscript submission, recruitment and data collection for the control group had already commenced. Recruitment for the control group started in July 2023 and treatment for this group was scheduled to be completed by May 2024 but was still ongoing. However, recruitment and data collection for the intervention group have not yet begun. These will start in July 2024. This protocol therefore reports procedures and analysis plans that were specified before completion of the intervention phase and before analysis of the main feasibility outcomes of the intervention group. The study is being conducted in accordance with version 1.2 of the ‘Aktiv im Alter’ study protocol from 27 April 2023, which has been reviewed by the ethics committees.

## Supplementary Information

Additional file 1: SPIRIT 2013 checklist: recommended items to be addressed in a clinical trial protocol and related documents.

Additional file 2: CONSORT 2010 checklist of information to include when reporting a pilot or feasibility trial.

## Data Availability

Not applicable. Data sharing is not applicable to this article as no datasets were generated or analysed.

## Abbreviations

DRKS: German Clinical Trials Register
FFB: Freiburg Physical Activity Questionnaire
FFbH: Hannover Functional Ability Questionnaire
GP: General practitioner
ICD-10: International Statistical Classification of Diseases and Related Health Problems, 10th revision
MRC: Medical Research Council
PA: Physical activity
PT: Physiotherapist
SF-8: Short Form Health Survey 8
SF-36: Short Form Health Survey 36
SoS: Social situation
UTAUT: Unified Theory for Acceptance and Use of Technology
SPIRIT: Standard Protocol Items: Recommendations for Interventional Trials
SPPB: Short Physical Performance Battery
*χ*^2^ test: Chi-square test
